# Microphysiological Conditions Do Not Affect MDR1-Mediated Transport of Rhodamine 123 above an Artificial Proximal Tubule

**DOI:** 10.3390/biomedicines11072045

**Published:** 2023-07-20

**Authors:** Negin Namazian Jam, Felix Gottlöber, Melanie Hempel, Yuliya Dzekhtsiarova, Stephan Behrens, Frank Sonntag, Jan Sradnick, Christian Hugo, Florian Schmieder

**Affiliations:** 1 Fraunhofer Institute for Material and Beam Technology IWS, 01277 Dresden, Germanystephan.behrens@iws.fraunhofer.de (S.B.); frank.sonntag@iws.fraunhofer.de (F.S.); 2 Department of Internal Medicine III, University Hospital Carl Gustav Carus, 01307 Dresden, Germany

**Keywords:** proximal tubule, multidrug resistance protein 1 (MDR1), transport kinetics, tariquidar, microphysiological systems, rhodamine 123, RPTEC/TERT1, apparent permeability

## Abstract

Despite disadvantages, such as high cost and their poor predictive value, animal experiments are still the state of the art for pharmaceutical substance testing. One reason for this problem is the inability of standard cell culture methods to emulate the physiological environment necessary to recapitulate in vivo processes. Microphysiological systems offer the opportunity to close this gap. In this study, we utilize a previously employed microphysiological system to examine the impact of pressure and flow on the transportation of substances mediated by multidrug resistance protein 1 (MDR1) across an artificial cell-based tubular barrier. By using a miniaturized fluorescence measurement device, we could continuously track the MDR1-mediated transport of rhodamine 123 above the artificial barrier over 48 h. We proved that applying pressure and flow affects both active and passive transport of rhodamine 123. Using experimental results and curve fittings, the kinetics of MDR1-mediated transport as well as passive transport were investigated; thus, a kinetic model that explains this transport above an artificial tubular barrier was identified. This kinetic model demonstrates that the simple Michaelis–Menten model is not an appropriate model to explain the MDR1-mediated transport; instead, Hill kinetics, with Hill slope of *n* = 2, is a better fit. The kinetic values, *K_m_*, *V_max_*, and apparent permeability (P_app_), obtained in this study are comparable with other in vivo and in vitro studies. Finally, the presented proximal tubule-on-a-chip can be used for pharmaceutical substance testing and to investigate pharmacokinetics of the renal transporter MDR1.

## 1. Introduction

In pharmaceutical substance testing, the interaction of potential drugs with different parts of the kidney is of major interest, as drug-induced kidney injuries are responsible for 19% of drug development failures in Phase III and 20% after approval [1]. Within the kidney, the proximal tubule is the first part to absorb essential organic solutes and ions, such as glucose, amino acids, and sodium, from the primary urinary filtrate. It also secretes xenobiotics and other metabolic waste products [2,3]. To manage secretion and absorption processes, the proximal tubular cells contain a broad range of about 400 membrane-based transporters that actively eliminate metabolic waste products and xenobiotics from the blood stream and absorb essential solutes from primary urine [4]. Within these transporters, multidrug resistance proteins (MDR) [5], also known as ABCB1 (ATP-binding cassette subfamily B member 1) transporters or P-glycoprotein (P-gp) and organic cation transporters (OCTs) [6], are of special interest because they are involved in the renal secretion of almost 30% of all prescribed drugs [4].

Therefore, the proximal tubule is the most relevant part in drug-induced kidney injury as well as drug–drug interactions. It is necessary to investigate the interaction of novel drugs with this part of the human kidney using advanced in vitro methods.

For several years, cell-based organ models have been employed for drug development [7,8], animal-free substance testing [9,10] and personalized medicine [11]. The cell-based organ models have advantages compared to in vivo methods. In vivo approaches are challenging due to the legislations and often have ethical issues due to animal testing [8,9]. The cell-based organ models, by contrast, do not suffer from such ethical issues and can be the future replacement for animal testing [9,10]. Ranging from static cell cultures in microwell plates to dynamic co-cultures in microphysiological systems (MPS), these cell-based assays allow the direct investigation of human cell layers, thereby helping to study the mechanisms of drug-induced kidney injury. Within the past decade, several two- and three-dimensional cell culture models of the kidney proximal tubule have been established [1,12,13,14,15]. Studies within these microphysiological environments have shown that proximal tubular cells stimulated by shear stress form dense monolayers and express an increased amount of ZO-1 [16] aquaporin and Na/K-ATPase [1] as well as α-smooth muscle actin (α-SMA) [14] compared to static culture conditions. Moreover, transporters that play an important role in drug-induced kidney injury, such as MDR-1, can be expressed within such microphysiological systems and their function can be impaired by ciclosporin A [15]. Nevertheless, key features of MDR1-mediated transport within such artificial tubular systems remain unaddressed. First, the microphysiological systems used to investigate MDR1 always apply a combination of flow and pressure, making it impossible to distinguish between these two independent factors. Second, flow is applied solely to induce fluid sheer stress. Since this leads to a false scaling of the volume flow, even in microphysiological systems, the complete effect of flow on MDR1-mediated transport remains unclear. Third, inhibition by ciclosporin A is not a real inhibition of the membrane-based transporter MDR1, but of cyclophilins [17,18], which play an important role in protein folding and cellular proliferation [19]. Fourth, time-resolved MDR1-mediated uptake or transport above artificial tubular systems to calculate the transport kinetics was not evaluated before. However, this is necessary to prove the predictive capability of microphysiological tubular systems.

**Figure 1:**
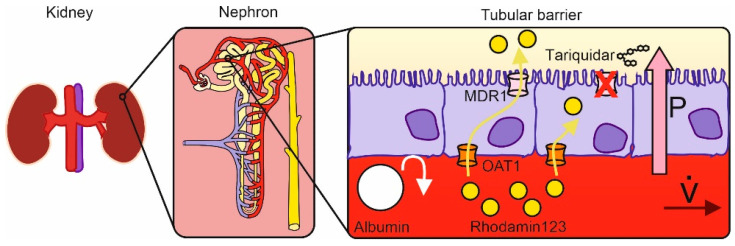
Graphical illustration of the proximal tubule model. The human kidney contains 1 million subunits known as nephrons. Each of these nephrons contains a proximal tubular part that is involved in the secretion of drugs from the peritubular capillary (shown in red) into the tubular lumen (shown in yellow). The arrow V shows the flow rate in the peritubular capillary. The cellular barrier between these two parts involves active transporters, such as MDR1 and organic anion transporter 1 (OAT1), carrying small molecules, such as rhodamin 123, from one side to the other. This study investigates the effect of pressure, flow, and selective inhibition on MDR1-mediated transport kinetics above an artificial tubular barrier.

Thus, the aim of the present study is to investigate the effects of microphysiological flow and pressure on MDR1-mediated molecular transport above an artificial cell-based model of the proximal tubule. Moreover, the kinetics of MDR1-mediated transport are characterized using the selective MDR1 inhibitor tariquidar [20] and time-resolved fluorescence measurements of rhodamine 123 (compare Figure 1).

## 2. Materials and Methods

### 2.1. Cell Culture (Barrier Formation)

Experiments were performed using an immortalized human renal proximal tubular epithelial cell line (RPTEC/TERT1) [21], which was supplied by Evercyte GmbH (Vienna, Austria). RPTEC/TERT1 is commonly used in in vitro and for similar assays. The immortalized cell line is chosen over primary cells due to the challenges of primary cells, like limited growth potential. To bring these cells into culture 0.6 × 10^5^ cells were seeded onto 24-well plates Thincert Transwell inserts (0.33 cm^2^, 0.4 µm pore size; Greiner Bio-one) and further cultured in ProxUp Basal Medium (Evercyte, Austria). The cells were cultured on Thincert inserts (Greiner Bio-one, Frickenhausen, Germany) for at least 8 days to ensure the formation of a confluent cell monolayer. The cell layer integrity was confirmed by measuring the trans-epithelial electrical resistance (TEER) with an epithelial volt-ohm-meter (EVOM, Millicel, Darmstadt, Germany). Transwell inserts were used for transport experiments when TEER values exceeded 150 Ω × cm^2^.

### 2.2. Transport Assay

Rhodamine 123 (R123) has been proven to be a substrate for MDR1. Due to its fluorescence and biochemical characteristics, R123 was chosen over other substrates of MDR1, like calcein AM, due to its low interference with underlying metabolic processes. Also, other MDR1 substrates have the risk of being transported by other ABC transporters, and not only just MDR1 but R123 is transported by MDR1 specifically [22]. Therefore, R123 was chosen as a substrate for transport assays. In addition, Alexafluor647 conjugated human serum albumin (HSA-647) was used as the negative control and to prove barrier integrity above the whole assay. The transport of R123 and HSA-647 across the tubular barrier was carried out under static and dynamic cultivation conditions. The static conditions were carried out by placing the Transwell^TM^ inserts on a well plate, with no pressure or flow applied. The dynamic experiments were in the microphysiological system (MPS), where microphysiological pressure and flow were applied; the dynamic conditions are explained in Section 2.3.

Fluorescence intensity at the beginning and end of the experiments was measured using a microplate reader (Tecan Infinite Pro, Tecan Group, Switzerland), and a miniaturized fluorescence reader (Fluo Sens Integrated, Qiagen, Hilden, Germany) was used for online monitoring. Fluorescence intensity of both R123 (excitation 505, emission 525 nm) and HSA647 (excitation 645, emission 671 nm) were measured every 2 min over 48 h. Before each experiment, fluorescence calibration curves of R123 and HSA647 were obtained with both readers to ensure the comparability of all results. Using the calibration curves, the fluorescent intensity (FU) results were converted to concentration (µM). 

**Figure 2:**
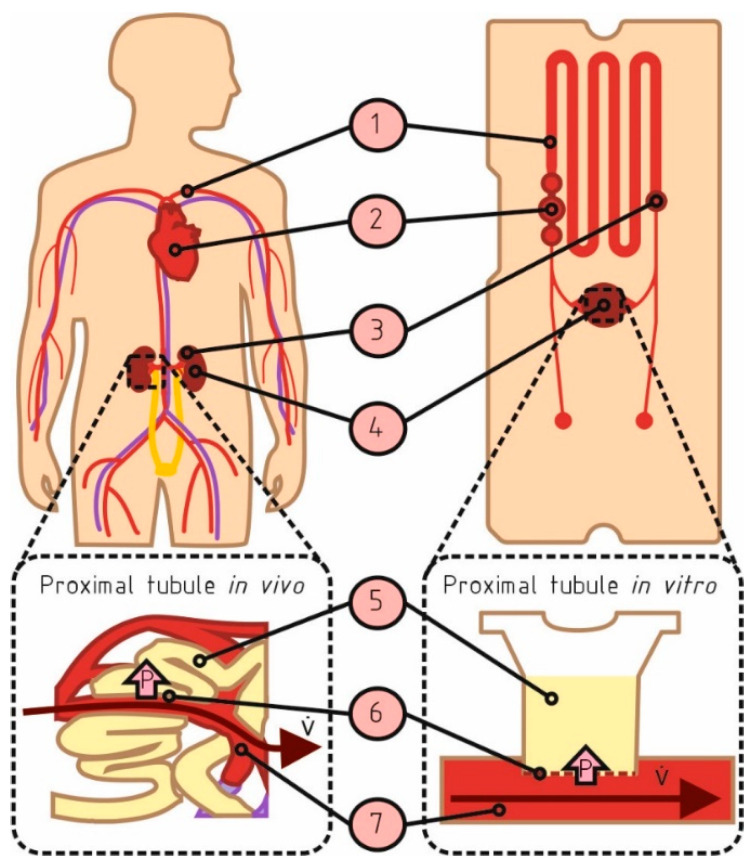
Tubular barrier within the microphysiological system (MPS). The physiological functions and their on-chip counterparts are shown: (1) blood vessel/microchannel; (2) heart/heart-like micropump; (3) autoregulation of kidney pressure by renin/angiotensin secretion/pressure port; (4) kidney with tubular lumen/artificial tubular compartment; (5) proximal tubule/artificial tubular compartment; (6) tubular barrier; (7) peritubular capillary/artificial peritubular capillary. The pink arrow (with P on it) indicates the direction of the pressure gradient P. The brown arrow (with V on it) indicates direction of flow with flow rate v̇.

During the transport assays, the decrease in the fluorescence intensity in the artificial microfluidic channels mimicking the peritubular capillary structures (in the well, see Figure 2; compartment 7) was measured constantly. R123 (2 μM) and HSA-647 (0.6 µM) were added to the cell culture medium and to the microfluidic channel (Figure 2), and a Transwell insert populated with cells (compartment 4 in Figure 2).

The whole system was then placed on the Qiagen fluorescence meter in such a way that only the fluorescence signal of the cell culture medium in the channels (compartment 1 in Figure 2) was measured every 120 s for maximum 48 h. Cell-free Transwell^TM^ inserts were used as a control. Transport assay experiments for each condition were performed and measured in triplicates.

To block the MDR1 activity, the specific noncompetitive inhibitor tariquidar (50 µg/mL [20]) was added into the cell culture media shortly before running the transport assay.

### 2.3. Adoption of the Microphysiological System

The microphysiological system (MPS [23]) was designed to mimic the microphysiological environment of either the glomerular or the tubular compartment of the human kidney. For this study, the MPS was used to mimic the microenvironment of the proximal tubular compartment (Figure 2).

The presented MPS consists of channels mimicking a blood circuit that connects a heart-like micropump and a cell culture compartment for the integration of the artificial tubular barrier (Figure 2). The MPS is designed to integrate a 24-well ThinCert™ cell culture insert containing a pre-cultured artificial proximal tubular barrier. The MPS was used to investigate two microphysiological aspects: flow and pressure. 

The dynamic experiments were performed using the MPS. After cells were grown to confluence (proved by TEER and microscopy), 24-well inserts were equipped with a sealing ring and placed into the insert-holder of the MPS. 

#### 2.3.1. Microphysiological Flow

To evaluate the physiological flow within the cell culture model on the MPS, the cellular surface area was used as a scaling factor. Compared to the tubular surface area of a single nephron (~1.76 mm^2^) [24], the cell culture insert covers the area of around 19 proximal tubules (33.6 mm^2^). For equal scaling of the flow for dynamic experiments, the integrated heart-like micropump [23] was set to a flow rate (v̇) of 0.13 μL/s, which is 19-fold of the estimated renal plasma flow into a single proximal tubule. It should be noted that flow and pressure can be adjusted independently. Nevertheless, when flow is applied, the integrated pneumatic pump generates a pulsatile pressure difference of ±5 mbar that corresponds to the frequency of the pump. Hence, this is further decreasing by the damping of the microfluidic system, and the average pressure is zero; it is neglected here. 

#### 2.3.2. Microphysiological Pressure

Pressure at the proximal tubular barrier was calculated and maintained according to the human in vivo environment. In general, the pressure at the proximal tubular barrier is the superposition of different pressure sources at both sides of the tubular barrier, as well as the pressure loss in the tubular interstitium [2]:

P = (P_cap_ + π_cap_) − (P_tub_ + π_tub_) − ΔP_int_ (1)

P_cap_—hydrodynamic pressure of the peritubular capillary;
P_tub_—hydrodynamic pressure of the proximal tubular lumen;
π_cap_—colloid osmotic pressure of the peritubular capillary;
π_bow_—colloid osmotic pressure of proximal tubular lumen;
ΔP_int_—pressure drop within the tubular interstitium.

While the capillary hydrodynamic pressure inside the peritubular capillary (Pcap) is ~10 mbar, the contrariwise hydrodynamic pressure inside the proximal tubular lumen is about 15 mbar. The colloid osmotic pressure inside the peritubular capillary is 35 mbar when leaving the glomerular capillaries [25]. When water is reabsorbed from the proximal tubular lumen, it decreases to 25 mbar at the end of the peritubular capillaries of the proximal tubule [2,25]. 

For the barrier model, this colloid osmotic pressure is set to 30 mbar. In contrast, there is no colloid osmotic pressure in the tubular lumen. Moreover, there is a pressure drop in the tubular interstitium consisting of a 10 mbar hydrostatic pressure drop and a 5 mbar colloid osmotic pressure drop [25]. Summing this up yields a pressure difference of 10 mbar pointing from the capillaries to the tubular lumen. In contrast to the in vivo situation, the cell culture media used in the tubular model system have no relevant colloid osmotic pressure, even after adding fluorescence-labeled albumin at the capillary side of the artificial GFB. Thus, the hydrodynamic pressure in the chip system was set to 10 mbar, consisting of 5 mbar hydrostatic pressure and 5 mbar applied via the pressure port (Figure 2; Nb. 3).

### 2.4. Fluorescence Microscopy

To check the MDR1 transporter activity, the RPTECs were stained with anti-MDR1 antibody (Abcam, Berlin, Germany) both under static and dynamic conditions. Transwell inserts covered with a confluent cell layer were stained according to a standard protocol [26]. When the cells were ready for microscopy, the membranes were cut from the Transwell inserts, placed on microscope glass slides, and covered with cover slips. Afterward, the samples were imaged using a BZ-9000 fluorescence microscope (Keyence, Osaka, Japan).

A cell viability kit (Merck, Darmstadt, Germany) consisting of Ethidium homodimer 1 (Eth-1) for dead cells and Calcein AM for live cells was used to evaluate the viability of the cell layer [26]. 

### 2.5. Quantitative Measurement of MDR1 Expression

To measure the amount of MDR1 expressed in different conditions, a sandwich enzyme-linked immunosorbent assay (ELISA) kit for MDR1 (antibodies-online, Aachen, Germany) was used. After each experiment, lysed cells were centrifuged at 5000× *g* for 5 min (at 4 °C); then, the supernatant was collected for ELISA [27]. For each experimental condition, three samples were prepared, with each sample measured in duplicates.

### 2.6. Statistical Analysis

The statistical analysis was performed using Minitab (Minitab^®^ Statistical Software, version 19.2020.2). For statistical tests, a two-tailed Student’s *t*-test/ANOVA for unpaired data was used. Kinetic fluorescence data were measured as triplicates and analyzed as time series; curve fits were generated using nonlinear regression.

All the curve fittings for kinetics and permeability investigation were performed using OriginPro2021b (version 9.85).

## 3. Results

### 3.1. Barrier Integrity of the Tubular Barrier

To characterize the barrier integrity of the artificial tubular barrier on the Transwell system, the cells were stained with calcein (living) and eth-1 (dead cells) as described in the Materials and Methods section. 

**Figure 3:**
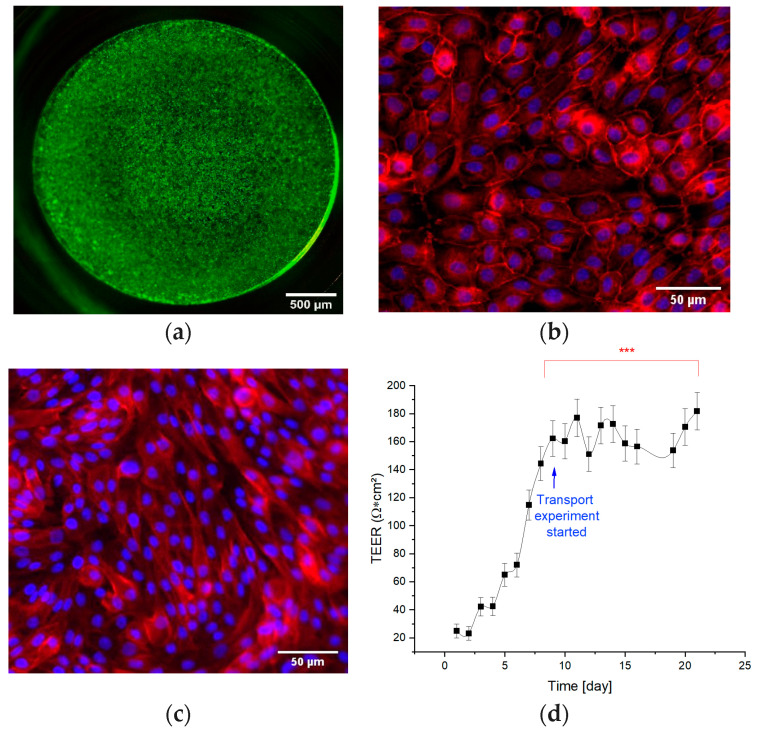
Barrier integrity: (**a**) live (green)–dead (red); (**b**) anti-E-cadherin (red), cell nuclei (blue, DAPI); (**c**) MDR1 expression, cell nuclei (DAPI, blue), and anti-MDR1 (red); (**d**) TEER measurements over 25 consecutive days. *** shows that the TEER values reached a plateau from day 8.

After live–dead staining, the confluence of the monolayer and a high viability were confirmed (Figure 3a). E-cadherin, which is important for the formation of junctions between cells, was observed (Figure 3b) and was located on the cell membrane as expected. The transport protein MDR1 was expressed in the cell membrane of RPTEC/TERT1 under static cultivation conditions (Figure 3c). Based on the immunofluorescence staining, it can be demonstrated that the RPTEC/TERT1 was positive for the anti-MDR1 under static cultivation conditions. The TEER value of the Transwell inserts was measured every two days to track the increase in the barrier integrity until a confluent cell layer was formed. As shown in Figure 3d, the cell layer became confluent after eight days of cultivation, by reaching a plateau in TEER values of about 150 Ω.cm^2^. At this time, flow and pressure experiments were started. For quality control the TEER value was also measured after each experiment to assess the confluence and tightness of the cell layer, by showing that it did not significantly decrease below 150 Ω.cm^2^.

### 3.2. Dynamic Cultivation

**Figure 4:**
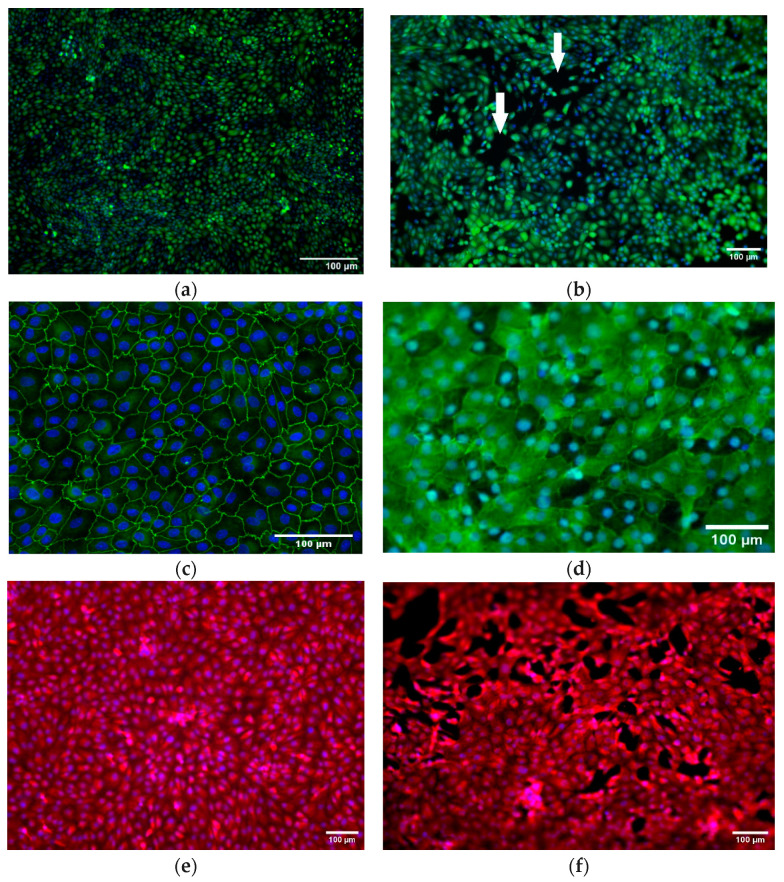
RPTEC/TERT1 seeded on Transwell inserts: (**a**) live–dead staining in static cultivation; (**b**) same insert shown in (**a**) after applying flow, white arrows show cavities; (**c**) anti-ZO-1 (green)- and DAPI (blue)-stained insert in static; (**d**) ZO-1 (green)- and DAPI (blue)-stained insert after flow experiment; (**e**) anti-MDR1 (red) and DAPI (blue) staining in static cultivation; (**f**) anti-MDR1 (red) and DAPI (blue) in dynamic cultivation, after 48 h of applying flow.

To investigate whether dynamic cultivation has any effect on the cell morphology immunofluorescence staining was used (Figure 4).

Comparing Figure 4a,b, which are live–dead images from the same insert before and after a flow experiment, we can see that the cell layer was more compact before, and applying flow caused some cavities in the cell layer. It should be noted that according to TEER measurements before and after applying flow, the cell layer’s confluence was not significantly affected, and TEER values remained at 150 Ω.cm^2^ (Figure 3d). Comparing the results from ZO-1 staining in (Figure 4c,d), there was no significant difference in morphology of the cells under static and flow conditions. This was confirmed by the cells’ area and diameter measurements using ImageJ (data shown in Supplementary Materials: Table S1). Figure 4c,d shows that in both static and dynamic cultivation, anti-ZO-1 was localized mostly on the membrane of the cells and at the tight junctions. MDR1 was expressed in both conditions (Figure 4e,f). A few cavities appeared after applying flow for 48 h (Figure 4b,f).

### 3.3. Time-Resolved Fluorescence Measurements of R123 Transport

To investigate the active molecular transport of R123, the fluorescence signal was measured under static and dynamic conditions. To constantly monitor the integrity of the cellular barrier, the fluorescence signal of HSA647 was measured simultaneously. In each condition, the transport was measured in the absence and presence of the MDR-1 blocker to study MDR1-mediated transport as well as passive transport (permeation of R123 through the cellular barrier). 

**Figure 5:**
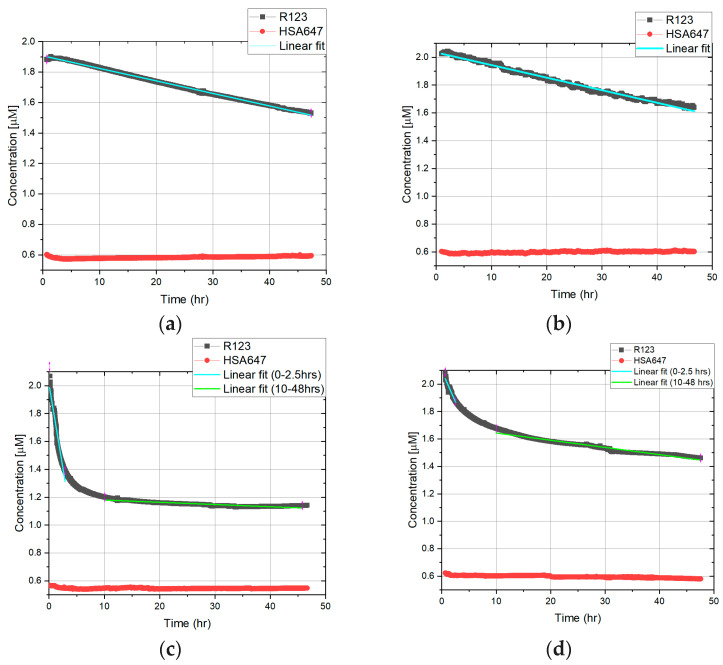

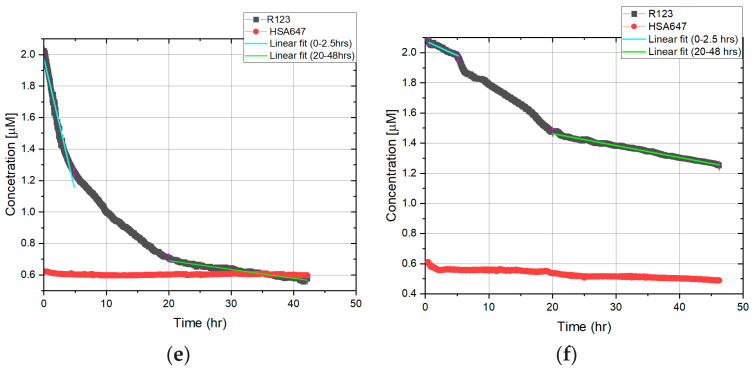
Online monitoring of a fluorescence signal in static and dynamic conditions. Experiments for each condition were conducted in triplicates. Slopes indicate the linearization in the mentioned time interval (explained in Section 3.4): (**a**) Static, no MDR1 inhibition; (**b**) Static, MDR1 inhibition; (**c**) 5 mbar pressure, no MDR1 inhibition; (**d**) 5 mbar pressure, MDR1 inhibition; (**e**) 0.13 µL/s flow, no MDR1 inhibition; (**f**) 0.13 µL/s flow, MDR1 inhibition.

As shown in Figure 5, under a static condition, the concentration of R123 in the peritubular capillary decreased to 1.5 µM over 48 h; however, when tariquidar was present, it decreased to 1.7 µM, showing that less R123 was transported to the tubular side. When 5 mbar pressure was applied, R123 concentration dropped to 1.1 µM from 1.5 µM, showing that more R123 was transported compared to the static condition. Therefore, a comparison of static and 5 mbar in the absence of tariquidar shows that 5 mbar pressure accelerated and increased the transport of R123. In addition, in the case of inhibited transport in pressure experiments, the transport took place slower with a smaller quantity. Interestingly, when flow was applied, the R123 concentration in the artificial peritubular capillary decreased dramatically to 0.6 µM, which shows that a significantly higher amount of R123 was transported in comparison with the static and 5 mbar experiments. The inhibition MDR-1-mediated transport can be seen in the flow experiments with tariquidar as well. Finally, Figure 5 shows the cellular barrier integrity, with a steady HSA647 fluorescence signal over 48 h in all of the experiments.

### 3.4. Permeability of the Barrier

Because concentration time curves do not serve as an easy-to-use indicator to describe the permeability, we were calculating the apparent permeability. In general, the apparent permeability $\left(P_{app}\right)$ is defined as the flow rate of a substrate through a biological barrier, normalized by the surface area and the initial donor concentration. $P_{app}\;$(cm/s) can be calculated using Equation (2):

(2) $$P_{app}=-\frac{dQ}{dt.\;A.\;C_{0}}$$

where d*Q*/d*t* is the solute flux (µmol/s) across the barrier, A is the surface area (cm^2^), and $C_{0}$ is the initial donor concentration (µmol/mL). $P_{app}$ is typically computed by adapting a straight line to the initial portion of the recorded amounts in the receiver compartment (here in the insert or artificial tubular lumen). Hence, this concept is just suitable for linear changes in the concentration in the receiver compartment; the online monitoring curves were fitted by linear regression. In the case of the static transport of R123 above the artificial tubular barrier, the transport process yielded a linear concentration time curve, and thus, the whole dataset from 0 to 48 h was used to extrapolate the slope (compare Figure 5a,b). In opposition to this pressure and flow dependent transport above the artificial barrier in two time intervals were used to extrapolate the slope. 

To have better understanding of the permeability of the barrier, the curves are fitted by linear function, and the slope of them is presented in Table 1. Linear fittings for HSA647 are presented in the Supplementary Materials, Tables S2 and S3.

**Figure 6:**
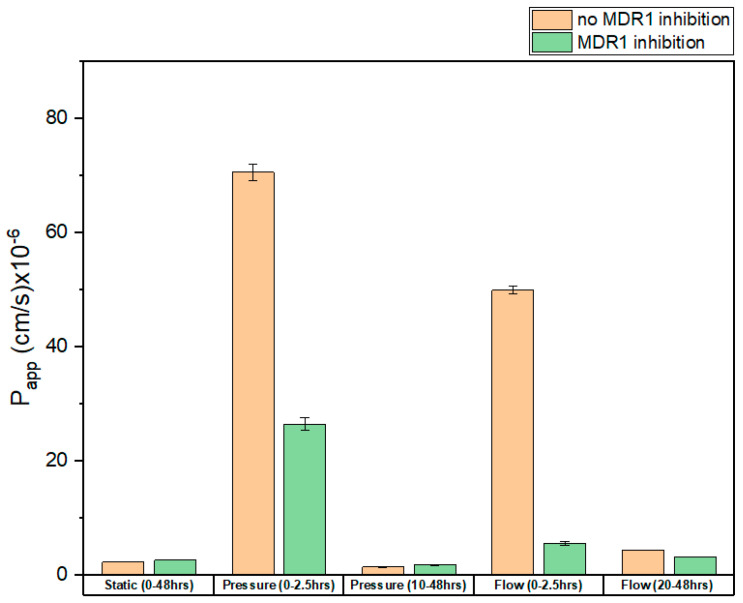
Apparent permeability of R123 through the barrier. The error bars designate the confidence intervals from the linear fittings.

In the results presented in Figure 5, the graphs and linear fittings are used to calculate the d*Q*/d*t* and therefore $P_{app}$. In the case of static conditions, only one $P_{app}$ is introduced since the trend of the transport is relatively steady. In the case of dynamic experiments, $P_{app}$ is introduced as two lines on the graph to describe it more precisely. Since the graphs in Figure 5 show the decrease in R123 concentration in the well (donor compartment), in the Equation (18), a minus is integrated to correspond to the direction of permeability. By having the values of d*Q*/d*t* from fittings and having *A* = 0.33 cm^2^ and *C*_0_ = 2 µM = 0.002 µmol/mL, *P_app_* is calculated as presented in Table 2 and Figure 6.

### 3.5. Quantitative Measurment of MDR1 Expression in Static and Dynamic Conditions

**Figure 7:**
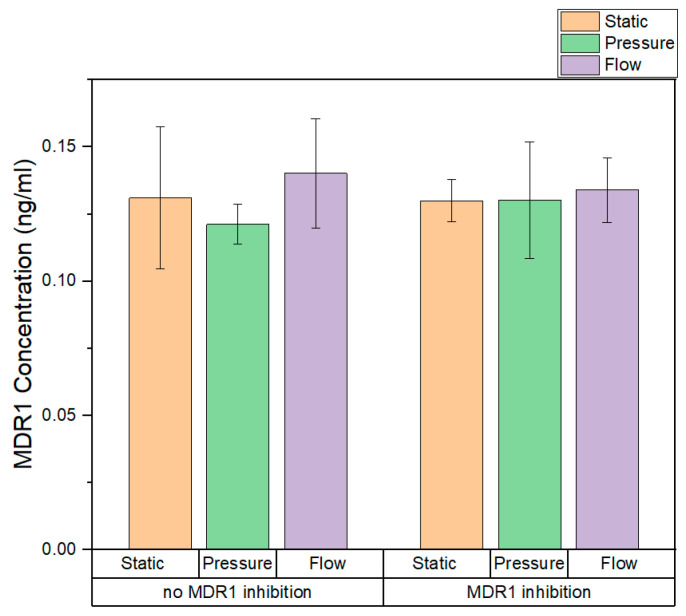
Amount of MDR1 expressed in different conditions. The values are shown as a mean, and the error bars are the standard deviation.

The amount of MDR1 expressed in the cell-based artificial proximal tubule was measured by the ELISA quantitative measurement. The results are shown in Figure 7.

The amount of MDR1 expressed by RPTECs in static and dynamic conditions was in the range of 0.12–0.14 ng/mL, and the values for dynamic cultivation do not differ from those of static cultivation. Nevertheless, flow and pressure did not affect the level of MDR1 protein by RPTECs. Furthermore, it can be seen that the presence of the inhibitor did not affect the level of MDR1 protein in each condition. To confirm, we performed the Student’s *t*-test; there is no significant difference in the mean values of MDR1 concentration in different conditions.

## 4. Discussion

In this study, a renal proximal tubular in vitro model was established, which is capable of secretion of the necessary transporter MDR1 (Figure 3c). 

We measured R123 transport in the well every two minutes for 48 h. In the presence and absence of the inhibitor, an active R123 transport was confirmed under static and dynamic conditions as well (Figure 5). In the presence of the MDR-1 inhibitor, the decrease in R123 concentration in the well was significantly reduced. 

Having a thorough understanding of pharmacokinetics is crucial when developing treatment strategies that incorporate medications [4,28]. Pharmacokinetics, as a discipline, strives to consolidate information about the distribution of drugs within the body and the effects of the body on the drug [4,28]. Therefore, we have also investigated the kinetics of R123 transport through the proximal tubule-on-a-chip.

### 4.1. Kinetic Characterization of Rhodamine Transport

R123 was used to evaluate the active and passive proximal tubular transport under artificial conditions. Active transport (AT) is a protein-mediated transport, while passive transport (PT) is when R123 molecules permeate through the artificial proximal tubular cell layer. In the presented system, a protein-mediated membrane transport starts with R123 molecules entering the cell through OAT1 [22] from the artificial peritubular capillary and then entering the tubular lumen through the MDR1 transporter (Figure 1).

Since the OAT1 is involved in the transport, the question is whether this will affect the kinetic model for R123 transport. To investigate the kinetics of an active transport, it is necessary to compare the K_m_ of both OAT1 and MDR1. Forster et al. [22] have studied the kinetics of MDR1- and OAT1-mediated transport of R123. For the present study, the simple Michaelis–Menten model was suggested, with the kinetic values for both protein transporters reported as K_m_ = 0.3 ± 0.1 and Vmax = 6.8 ± 0.5 µM for OAT1 and K_m_ = 17.5 ± 2.8, Vmax = 20.4 ± 1.4 µM for MDR1-mediated transport [4,22]. This means that the MDR1-mediated efflux is much faster and more effective than the OAT1-mediated influx; consequently, the amount of R123 within the cells is negligible [22]. Therefore, in the kinetic model, the kinetic values of OAT1-mediated transport will be neglected [22,29].

#### 4.1.1. Kinetic Characterization of R123 Transport—Static Conditions

To find the correct kinetic model, experimental measurements as well as computer simulations have been performed in other studies [22,28,29,30]. Many studies suggest that the simple steady-state Michaelis–Menten model does not explain the protein-mediated membrane transfer by MDR1 [22,28,29,30]. They suggest that there is a cooperative behavior in R123 transport by MDR1; therefore, the Hill kinetics with a Hill slope of 2 (*n* = 2) best fit the transport mechanism of MDR1, as shown below [22,28,29,30].

(3) $$V=\frac{V_{max}{\left[S\right]}^{n}}{K_{m}{}^{n}+{\left[S\right]}^{n}}$$

In this equation, *V* is the substrate transport velocity in static conditions, [S] is the substrate (R123) concentration, *V_max_* is the maximum velocity of R123 transport, *K_m_* is Michaelis–Menten constant, and *n* is the Hill slope or degree, which usually explains the cooperative behavior in substance transport. It should be noted that, in this definition, *K_m_* = [S] when *V* = 0.5 *V_max_*.

**Figure 8:**
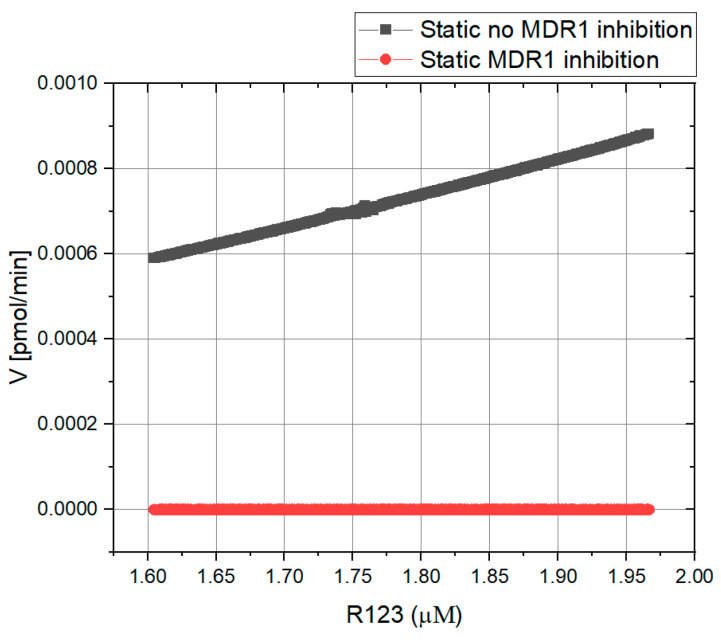
Rate of R123 transport: MDR1-mediated transport (active transport, gray) and passive permeation (red). The rate of R123 transport in absence of tariquidar is shown in gray. The amount of passively permeated R123 can be measured in the presence of an inhibitor (red). Both curves were fitted to Hill kinetics with R^2^ ≥ 0.98 using OriginPro2021 software.

To prove that the presented system follows the Hill kinetics, the *V* vs. [S] curve (Figure 8) was calculated using the R123 concentration from the time-resolved fluorescence measurements using *n* = 2, *K_m_* = 17.5 µM and *V_max_* = 0.0525 pmol/min from the literature [4,22,28,29,30]. The curve was fitted to the Hill model using OriginPro2021 software, with R^2^ = 0.99, and the kinetic values *K_m_* = 17.47 ± 8 µM & *V_max_* = 0.069 ± 0.05 pmol/min were obtained, which are very close to those from the literature (see Table 3) [22,28].

To determine the passive permeation of R123, the non-competitive inhibitor tariquidar was added to the proximal tubule compartment. It is assumed that when the inhibitor is present, any R123 molecule that passed through the membrane passed by means of passive transport [29]. Therefore, the measurement in the presence of the inhibitor shows the amount of R123 that permeated through the membrane. To calculate the kinetic values in the presence of tariquidar, the *V* (rate of R123 transport) vs. [S] (R123 concentration) curve was plotted (Figure 8) using online monitoring results, providing the R123 concentration. Tariquidar is a non-competitive inhibitor [20]; thus, *Km* remains the same (17.5 µM), and *V_max_* is decreased and calculated from the equation below:

(4) $$V_{max}^{\prime }=\frac{\;V_{max}}{\left(1+\frac{\left[I\right]}{K_{I}}\right)}$$

where $V_{max}^{\prime }$ is the maximum velocity in the presence of tariquidar. [I] is the concentration of the inhibitor, and K_I_ is the inhibition constant of tariquidar in interaction with MDR1. With *K_I_* = 0.32 nM [32], $V_{max}^{\prime }$ = 2.18 × 10^−7^ pmol/min was calculated.

The same procedure was performed in the presence of tariquidar considering the new value for *V_max_*. *K_m_* = 11.06 ± 0.25 µM and *V_max_* = 1.25 × 10^−7^ ± 3 × 10^−9^ were calculated from the curve, and the curve was fitted to the Hill kinetics with R^2^ = 0.99, proving that even in the case of inhibition, the Hill kinetics fit the presented system. The kinetic values are in the range of those in the literature (Table 4) [22,32]. As mentioned previously, the rate of transported R123 in the presence of tariquidar is considered to be the passive transport rate, which is very low (Figure 8). This is corresponding to an intact cell culture model and, moreover, proves that almost no R123 is passively transported through the intercellular space.

Thus, the R123 transport kinetics in the static condition can be described by the following two equations:

(5) $$J_{\begin{array}{c} static\\ AT \end{array}}=\frac{V_{max}{\left[S\right]}^{n}}{K_{m}{}^{n}+{\left[S\right]}^{n}}$$

(6) $$J_{\begin{array}{c} static\\ PT \end{array}}=P\times C_{0}$$

where $J_{\begin{array}{c} static\\ AT \end{array}}\;$ is active transport (MDR1-mediated transport) in the static conditions and follows Hill kinetics. $J_{\begin{array}{c} static\\ PT \end{array}}$ describes the passive transport in static conditions, where P is the passive permeability of the substrate and $C_{0}$ is the initial concentration of the substrate. After $J_{\begin{array}{c} static\\ PT \end{array}}$ was measured as 0.030 pmol/min, *P* could be calculated from Equation (5) as 1.5 × 10^−8^ L/min. If P is further divided by the surface area of 0.33 cm^2^, it is comparable to the calculated value of *P_app_* from Table 2 at static conditions and in the presence of an inhibitor.

#### 4.1.2. The Kinetic Characterization of R123 Transport—Dynamic Conditions

The next step is to establish a model that can describe the effect of flow and/or pressure on permeation.

First, the effect of pressure is discussed. To describe how pressure affects the permeation, Kedem–Katchsalsky equations and Darcy’s law were used. Kedem–Katchsalsky equations, which are also known as the revised Starling principle, explain that the net fluid flux across a capillary wall is proportional to the net driving force, or the so-called Starling forces [33,34,35,36,37]:

(7) $$J_{v}=L_{p}\left(\Delta P-\sigma \Delta \pi \right)=L_{p}\Delta P-L_{p}\sigma \Delta \pi$$

where $J_{v}$ is the volume flow and $L_{p}$ is the hydraulic conductivity (also known as the membrane filtration coefficient) from Darcy’s law. Equation (7) characterizes both the porous medium and the liquid, with Δπ denoting the osmotic pressure difference, and ΔP denoting the hydrostatic pressure difference. Peritubular Starling forces have an important role in the regulation of proximal tubular solute transport [38]. It should be noted that Equation (7) only describes the effect of Starling forces (hydrostatic pressure and osmotic pressure) on passive transport; therefore, for the calculations, the values from experiments in the presence of tariquidar are used.

Investigating the effect of pressure on R123 flux requires coupling the solvent flux ($J_{s}$)—that is, the R123 flux—to the volume flow ($J_{v}$) across the porous membrane. For this purpose, the second Kedem–Katchsalsky equation can be used [33,34,35,36,39]:

(8) $$J_{s}=J_{v}\bar{c}\left(1-\sigma_{s}\right)+\omega \Delta \pi$$

where $\sigma_{s}$ is the reflection coefficient, which is a measure of the relationship between the permeability of the solute and that of the water. ω is the solute permeability, and $\bar{c}\;$ is the mean concentration of both sides of the membrane: $\bar{c}=\frac{1}{2}\left(c_{1}+c_{2}\right)\;$ [33,34,35,39].

Equations (7) and (8) have been widely used in research on non-electrolyte substance permeability through artificial and biological membranes [34,35,39]. 

In the MPS used here, the difference in osmotic pressure is measured in the presence of 2 µM R123 and with no R123 as a control in the ProxUp media. According to this measurement, Δπ≅0. Considering that the effects of pressure and flow are investigated separately, $J_{s}$ will be written from here on as $J_{R123,P,\;PT}$ for pressure-mediated passive transport and $J_{R123,F,\;PT}$ for flow-induced passive transport. Therefore, for pressure-mediated transport, we can re-write Equations (7) and (8) as follows:

(9) $$J_{v}=L_{p}\Delta P$$

(10) $$J_{R123,P,\;PT}=J_{v}\bar{c}\left(1-\sigma \right)$$

Combining Equations (9) and (10), we can write

(11) $$J_{R123,P,PT}=L_{p}\Delta P\bar{c}\left(1-\sigma \right)$$

$L_{p}$ was measured in a set of experiments, where the volume flow was measured (*n* = 9) at different pressure differences (5, 10, 30 mbar). Then, using Equation (9), the value of $L_{p}$ was obtained:

$$L_{p}=\frac{J_{v}\;\left[\mu L{.\min}^{-1}{.\mathrm{cm}}^{-2}\right]}{\Delta P\;\left[\mathrm{mbar}\right]}=0.028\pm 0.002\;\frac{\mu L}{\min{.\mathrm{cm}}^{2}.\mathrm{mbar}}$$

The reflection coefficient σ was defined by Staverman. He demonstrated that σ = 1 for an impermeable solute and σ < 1 for a permeable solute [33]. Since the cell layer is impermeable for albumin, $\sigma_{Alb}≅1$ and, therefore, $J_{s_{Alb}}≅0$. The membrane is semi-permeable to rhodamine 123 (the solute); $\sigma_{R123}<1$,$\;J_{s_{R123}}>0$.

Measuring the reflection coefficient of a specific solute is not easy, but the sieving coefficient is usually measured and presented in clinical studies; therefore, it is a better parameter to use in equations. Chen et al. have shown that the reflection coefficient can be estimated using the sieving coefficient by subtracting the sieving coefficient (θ) from 1 [40]:

(12) σ≅1−θ

The sieving coefficient (θ) in our system can be calculated by dividing the concentration of R123 in the insert over its concentration in the well. These values can be obtained from the experimental results. In the pressure experiments, the concentration in the well and the insert were measured in four Transwell inserts after 48 h; the value of the sieving coefficient and reflection coefficient were obtained as θ=0.22 & σ=0.78.

With values of $L_{p}$ and  σ, the amount of passively permeated R123 affected by microphysiological pressure (ΔP=5 mbar) can be estimated using Equation (11): $J_{R123,P,PT}=0.0102\frac{pmol}{min}$.

Nevertheless, the real value of $J_{R123,P,PT}$ was calculated using experimental results from online monitoring and end-point measurements; it is equal to 0.027 $\frac{pmol}{min}$. The predicted value and the actual amount of R123 that was transported passively are very close, showing that the suggested equations can be used to estimate the passive transport of R123 affected by pressure.

In this study, the effect of flow on R123 transport was also investigated. The next step is to establish an equation that can describe the effect of flow on the passive permeation of R123. For this purpose, Equation (9) can be re-written in this form [41]:

(13) $$J_{v}=\frac{\Delta V}{A\;\Delta t}$$

where *A* is the area of the possible permeation and $\frac{\Delta V}{\Delta t}$ is the volume flow passing through the membrane over time. Then, combining Equations (10) and (13), we obtain

(14) $$J_{R123,F,PT}=J_{v}\bar{c}\left(1-\sigma \right)=\frac{\Delta V}{A\;\Delta t}\bar{c}\left(1-\sigma \right)$$

In this case, σ is different since the sieving coefficient is affected; therefore, it was re-calculated using the data from flow experiments: σ = 0.51.

To predict the effect of flow on the amount of R123 transported passively, $\frac{\Delta V}{\Delta t}$ must be calculated. The flow rate in the MPS underneath the artificial proximal tubule was set to 0.13 µL/s, but this flow rate is different from the actual $\frac{\Delta V}{\Delta t}$ that passed through the membrane. Nevertheless, $\frac{\Delta V}{\Delta t}=0.052\;\mu L/\min$ was calculated by measuring the amount of liquid that passed through the membrane within two days. Using these values, the amount of R123 that will permeate passively when affected by flow can be predicted using Equation (14): $J_{R123,F,PT}=0.026\;\frac{\mathrm{pmol}}{\min}$. Compared to this the actual amount of R123 from endpoint measurements was 0.064 $\frac{\mathrm{pmol}}{\min}$.

All in all, the total passively transported R123 can be calculated as below:

(15) $$J_{R123,PT}=J_{R123,static,PT}+J_{R123,P,PT}+J_{R123,F,PT}$$

(16) $$J_{R123,PT}=P\times C_{0}+SL_{p}\Delta P\bar{c}\left(1-\sigma_{s}\right)+\frac{\Delta V}{A\;\Delta t}\bar{c}\left(1-\sigma_{s}\right)$$

The next step to characterizing R123 transport is to investigate whether flow and pressure affect MDR1-mediated transport. For this purpose, it is necessary to calculate the effect of pressure and flow on the transport of R123 (($R_{diff_{pressure}}$) and ($R_{diff_{flow}}$), respectively) compared to the static conditions. We can write these as Equations (17) and (18).

(17) $$J_{R123_{\;flow}}=J_{R123_{\;static}}+R_{diff_{flow}}$$

(18) $$J_{R123_{\;pressure}}=J_{R123_{\;static\;}}+R_{diff_{pressure}}$$

The total transport (including both passive and active transport in the absence of an inhibitor) affected by flow ($J_{R123_{\;flow}}$) is measured using the endpoints from the flow experiments. In the absence of the inhibitor, the average rate of transport was 0.112 pmol/min. Therefore, $R_{diff_{flow}}=0.0434pmol/min$. The same applies for pressure experiments; based on the endpoints in the pressure experiments in absence of the inhibitor, the average rate of transport was 0.069 pmol/min; therefore, $R_{diff_{pressure}}=0.0009\;pmol/min$, which is negligible. Since flow had no significant effect on the amount of expressed MDR1 (Section 3.5), this increase in transport is mediated by two main effects. First, flow itself increases the rate of molecules actively transported by MDR1. This change can be explained by the increased reuptake of R123 that is driven by convection. Second, by applying 0.13 µL/s flow, small cavities in between the cells appear after 48 h (Figure 4b,f). Nevertheless, since the TEER values still do not significantly decrease, the barrier seems intact. However, these cavities can explain the increased transport in the flow experiment and the non-negligible $R_{diff_{flow}}$.

By having the passive transport rates (in the presence of the inhibitor) from Table 5 and having the total transport rates (in the absence of the inhibitor) from Table 6, the active transport rates are calculated and summarized in Table 7. The values for active transport of R123 at different conditions are in the same range, perfectly showing that the dynamic conditions did not affect the active transport itself but affected the passive transport and therefore the total transport.

### 4.2. Apparent Permeability Coefficients

We obtained the apparent permeability coefficients (*P_app_*) of R123 for each condition. As shown in Figure 6, the dynamic conditions increased the apparent permeability. The 5 mbar pressure led to a dramatic increase in apparent permeability in the first 2.5 h. In the absence of an inhibitor, it is 30 times higher compared to static conditions. Surprisingly, in the presence of an inhibitor, *P_app_* was increased 10-fold upon 5 mbar pressure. In addition, flow had the same effect, and the *P_app_* was higher: 25-fold and 2-fold in the absence and presence of the MDR-1 inhibitor, respectively. Therefore, these results show that dynamic condition increases the apparent permeability. In the second measurement interval, the *P_app_* was lower compared to the first hours, which was expected, since the concentration of R123 is increased on the receiver compartment and the ΔC is lower compared to the first measurement interval.

The values obtained for *P_app_* agree with those from the literature [42,43]. Fortuna et al. [42] studied the apparent permeability of the MDR1 transporter in mouse small intestine, where they studied the transport of R123 as well as other drugs with a relatively similar size to R123, such as ciprofloxacin by MDR1. Our *P_app_* values match those of their study. In another study, Larson et al. [43] studied the apparent permeability of R123 through MDR1 in both the Madin–Darby canine kidney cells monolayer and the CaCO_2_-cells monolayer, where they show similar values for *P_app_*. Thus, the presented proximal tubule-on-a-chip shows the capability to investigate the permeability and efflux of drugs by MDR1. 

## 5. Conclusions

In the current study, we investigated the effects of microphysiological flow and pressure on the MDR1-mediated transport of R123, as well as on its passive transport, in a model of the proximal tubule in vitro. 

Firstly, we created an artificial cell-based model of the proximal tubule within a microphysiological system that can mimic the MDR1-mediated transport of R123 within physiological and non-physiological conditions. Based on this model, we investigated the effect of pressure and flow on the transport of rhodamine above the artificial proximal tubule.

As a negative control, we characterized the kinetics of MDR1-mediated transport using time-resolved fluorescence measurements of R123 in the absence and presence of tariquidar, an MDR1 inhibitor, but without pressure or flow. The results show that the passive transport of R123 (MDR1 blocked by tariquidar) in static conditions was negligible. This correlates with a dense and confluent cell layer that is almost impermeable for small molecules, even in the intercellular space. Thus, it closely recapitulates the in vivo situation and can therefore be used as a model system for tubular reabsorption.

Based on the static transport values, we investigated the effect of pressure and flow on the transport. Our results prove that pressure and flow affect the transport of R123 from the artificial peritubular capillary to the tubular compartment. A pressure of 5 mbar increases the transport and the apparent permeability, but not significantly. Also, vertical flow underneath the artificial proximal tubule of 0.13 µL/s had a significant effect on transport, demonstrated by the accelerated transport yielding in a higher quantity of transported R123 in the artificial tubular lumen (Figure 5). Comparing the effects of physiological pressure (5 mbar) and flow (0.13 µL/s), flow had a significantly larger effect on the transport. Our results agree with previous studies that showed that dynamic conditions, such as flow, can improve the function of ABCB1 transporters, such as MDR1 [44]. However, in some previous studies, it was reported that applying flow causing even very low shear stress can change the morphology of proximal tubule cells [16]. This effect was not observed in our case. However, it should be noted that, in our system, the proximal tubular cell layer was not directly affected by shear stress induced by the flow, since the cells are on top of the insert, which was not directly exposed to shear stress. Nevertheless, the cells experience some stress from the flow underneath them; additionally, the pulsatile flow causes the artificial proximal tubule to deflect. This deflection is also considered to affect the transport of R123 in both active and passive pathways, but we could not observe any effect on cell morphology [16].

To differentiate between the passive and active transport above the barrier under static and dynamic conditions, we created a mathematical model to describe the kinetic processes affecting the transport. The model was based on Hill kinetics that closely reflects the MDR1-mediated transport and is superior to the simpler Michaelis–Menten kinetics.

In static conditions, we showed that transport follows Hill kinetics with a slope of *n* = 2 in the presence and absence of the MDR1 inhibitor. This agrees with previous studies that showed that MDR1 has multiple co-operative binding sites where ligands, including R123, can be transported [22,28,30,31]. The calculated kinetic parameters from our measurements (*V_max_*, *K_m_* and *n*) under static conditions agree with those from other studies [4,22,28,29]. Nevertheless, to our knowledge this was the first study to prove this for a renal tubular barrier model. This is important; hence, barrier models mimic the peritubular capillary and the tubular lumen and thus create a polarization of the tubular cells. From this point of view, it is interesting to highlight that this type of culture does not influence the kinetic characteristics of MDR1-mediated R123 transport compared to adherent culture conditions [22,28].

To describe the transport of R123 in dynamic conditions, a combination of Darcy’s law and a revised Starling’s equation (Kedem–Katchlasky) [28,29,34] were used. The rates of passive transport in the experiments fit to the values predicted by the aforementioned Equations (11) and (14), confirming that these equations effectively describe the transport within the system. Using the aforementioned equations, we could describe the effect of flow and pressure on both the passive and active transport. 

During drug development, the renal transporters, such as MDR1, must be evaluated due to their key role of drug transport and reuptake [4]. The presented kinetic characterization, and its agreement with other studies, shows that this system is highly useful for further studies and for investigating the pharmacokinetics of novel assay developing drugs. Also, the kinetic model presented here can be used for barrier models to have a better understanding of the effects of the treatment of human proximal tubules. 

We expect the model to have a different performance for lipophilic and hydrophobic components or if a substrate with different molecular size is used. This needs further investigation in future studies. 

For the first time, we established a kinetic model for active and passive transport of R123 in both static and dynamic conditions in an artificial renal proximal tubular barrier model. We have shown that this MPS can be used in future studies to investigate other aspects of MDR1-mediated substance transport, nephrotoxicity, and interactions of the tubular human kidney compartment in vitro.

## Figures and Tables

**Table 1:** Slope of the fitted lines of the R123 curve of Figure 5. The values are shown with confidence intervals from the fitting.

	No MDR-1 Inhibition		MDR-1 Inhibition	
Condition	Slope	R-Square	Slope	R-Square
Static	−0.008 ± 0.0001	0.99	−0.009 ± 0.00002	0.98
Pressure (0–2.5 h)	−0.24 ± 0.005	0.92	−0.09 ± 0.0038	0.91
Pressure (10–48 h)	−0.002 ± 0.0002	0.91	−0.005 ± 0.00003	0.96
Flow (0–2.5 h)	−0.17 ± 0.002	0.96	−0.017 ± 0.0009	0.97
Flow (20–48 h)	−0.006 ± 0.0001	0.97	−0.008 ± 0.0001	0.99

**Table 2:** Apparent permeability of R123. The values are shown with confidence intervals from the fitting.

	Papp (cm/s) × 10^− 6^	
Condition	No MDR1 Inhibition	MDR1 Inhibition
Static	2.35 ± 0.003	2.65 ± 0.01
Pressure (0−2.5 h)	70.55 ± 1.47	26.45 ± 1.12
Pressure (10−48 h)	1.43 ± 0.10	1.83 ± 0.11
Flow (0−2.5 h)	49.97 ± 0.59	5.58 ± 0.06
Flow (20−48 h)	4.41 ± 0.06	3.22 ± 0.04

**Table 3:** Comparison of kinetic parameters in the absence of an inhibitor at static conditions between the literature and the present study.

	K_m_ (μM)	V_max_ (pmol/min)	n
From the literature	≅17.5 [4,22,28,31]	0.0525 [22]	2 [16,22,28]
Obtained values (present study)	17.47 ± 8	0.069 ± 0.05	2

**Table 4:** Comparison of kinetic parameters in the presence of an inhibitor at static conditions between the literature and the present study.

	K_m_ (μM)	V^’^_max_ (pmol/min)	n
From the literature	≅17.5 [4,22,28,31]	2.18 × 10^−7^ (from Equation (3)) [32]	2 [22,28]
Obtained values (present study)	11.06 ± 0.25	1.25 × 10^−7^ ± 3 × 10^−9^ (from curve fitting)	2

**Table 5:** Passive transport of R123.

Conditions of Passive Transport of R123	Symbols	Calculated from Equation $\left(\frac{pmol}{min}\right)$	Measured $\left(\frac{pmol}{min}\right)$
Pressure	$J_{R123,P,PT}$	0.0102	0.027
Flow	$J_{R123,F,PT}$	0.026	0.064

**Table 6:** Total transport of R123.

Conditions of Total Transport Rate of R123	Symbol	Measured $\left(\frac{pmol}{min}\right)$
Static	$J_{R123_{static}}$	0.068
Pressure	$J_{R123_{pressure}}$	0.069
Flow	$J_{R123_{flow}}$	0.112

**Table 7:** Active transport of R123.

Conditions of Active Transport Rate of R123	Symbol	Measured $\left(\frac{pmol}{min}\right)$
Static	$J_{R123_{\mathrm{AT},static}}$	0.038
Pressure	$J_{R123_{\mathrm{AT},pressure}}$	0.042
Flow	$J_{R123_{\mathrm{AT},flow}}$	0.048

## Data Availability

Not applicable.

## Supplementary Materials

The following supporting information can be downloaded at: https://www.mdpi.com/article/10.3390/biomedicines11072045/s1, Table S1: cells area measurements under static and flow conditions; Figure S1: Linear fittings for R123 and HSA647 transport; Table S2: slope of the fitted lines of HSA647 curve of Figure 5. Table S3: apparent permeability of HSA647. Table S4: fluorescence Endpoints after 48 hours of experiment.
