# Supplementary material for: Microphysiological Conditions Do Not Affect MDR1-Mediated Transport of Rhodamine 123 above an Artificial Proximal Tubule
Source: Biomedicines. 2023 Jul 20;11(7):2045. doi: 10.3390/biomedicines11072045 (PMC10376999; doi:10.3390/biomedicines11072045)
Supplement: Supplementary file 1 [file biomedicines-11-02045-s001.zip › biomedicines-2508549-SI.pdf]

Supplementary material

Microphysiological Conditions Do Not Affect MDR1-Mediated Transport of Rhodamine 123 above an Artificial Proximal Tubule

Negin Namazian Jam [1], Felix Gottlöber [1], Melanie Hempel [1], Yuliya Dzekhtsiarova [1], Stephan Behrens [1], Frank Sonntag [1], Jan Sradnick [2], Christian Hugo [2] and Florian Schmieder [1,*]

Morphology of the cells after applying flow

The area of the cells under static and flow conditions was measured by analyze particles tool in the ImageJ software. The results are presented in table S01. These results were checked for significance difference by applying a T-test (OriginPro2021), which showed no significance difference in the area of the cells.

Table S1- cells area measurements under static and flow conditions

	Area	
	static	flow
1	710.874	447.704
2	626.499	733.833
3	487.596	882.781
4	165.306	208.642
5	427.902	692.219
6	527.2	989.828
7	709.439	428.763
8	346.684	724.649
9	223.278	229.879
10	611.575	576.276
11	614.158	701.977
12	234.184	635.395
13	442.825	1142.506
14	1016.518	684.758
15	409.247	418.431
16	577.71	609.853
17	494.77	406.091
18	321.429	409.821
19	465.21	165.019
20	756.218	474.681
21	434.216	498.788
22	443.112	608.992
23	728.954	974.904
24	544.994	431.346
25	485.013	274.936
26	462.341	518.017
27	639.413	795.249
28	517.73	518.878
29	634.534	568.814

30	1133.323	643.718
31	500.797	363.329
32	531.792	590.051
33	243.08	658.642
34	397.768	835.14
35	212.946	150.957
36	764.254	637.978
37	896.556	560.491
38	369.643	697.385
39	544.707	545.281
40	1089.126	150.957
41	588.042	642.57
42	421.875	726.371
43	327.742	438.807
44	481.282	462.341
45	688.776	493.335
46	344.675	544.133
47	408.96	294.452
48	293.304	394.611
49	432.494	442.251
50	549.298	543.846

HSA647 fitting lines and Papp

Figure S1- Linear fittings for R123 and HSA647 transport

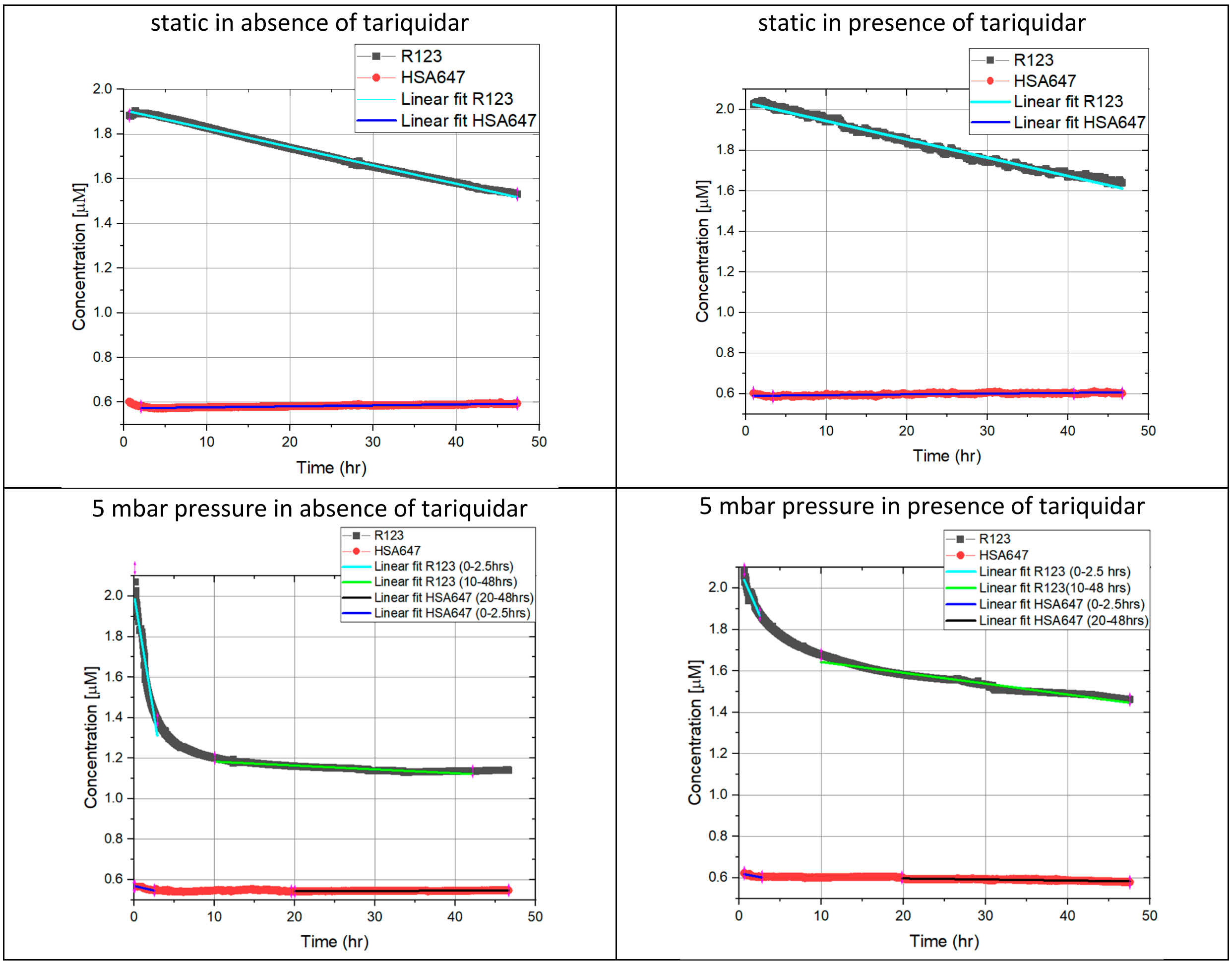

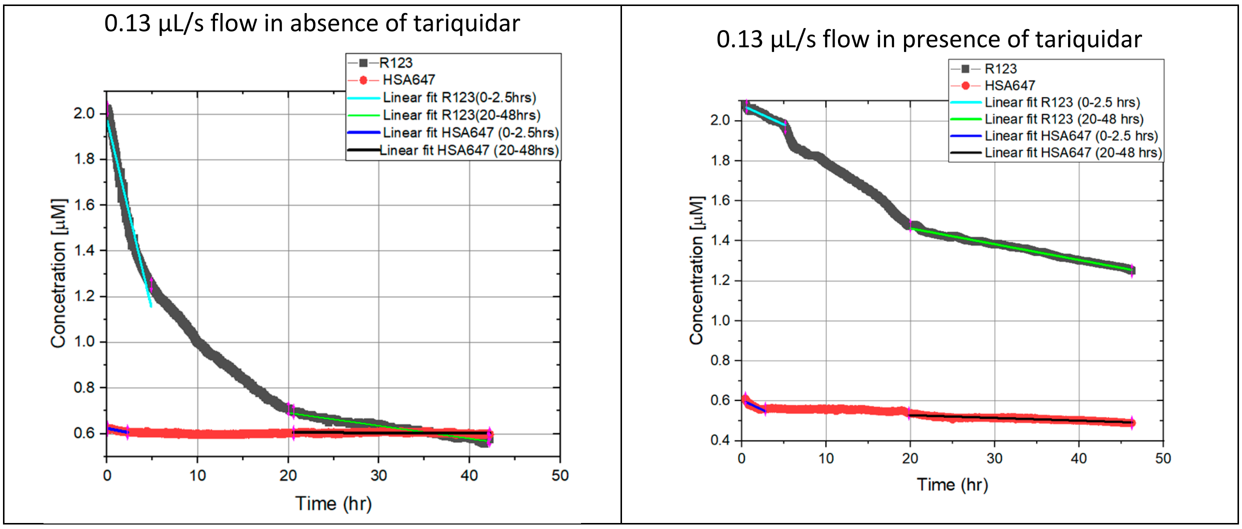

Table S2- slope of the fitted lines of HSA647 curve of figure 5. The errors are confidence intervals for the fitting.

	no MDR-1 inhibition		MDR-1 inhibition	
Condition	slope	R-square	Slope	R-square
Static	$-4.32\times10^{-4}\pm2.7\times10^{-6}$	0.95	$3.66\times10^{-4}\pm7.3\times10^{-6}$	0.65
Pressure (0-2.5hrs)	$-0.0092\pm2.63\times10^{-4}$	0.91	$-0.007\pm5.27\times10^{-4}$	0.76
Pressure (20-48hrs)	$-1.47\times10^{-4}\pm2.71\times10^{-6}$	0.62	$-5.01\times10^{-4}\pm1\times10^{-5}$	0.76
Flow (0-2.5hrs)	$-0.005\pm2.2\times10^{-4}$	0.94	$-0.0014\pm1.48\times10^{-5}$	0.89
Flow (20-48hrs)	$-1.61\times10^{-4}\pm1.8\times10^{-5}$	0.64	$-0.001\pm1.48\times10^{-5}$	0.92

Table S3- apparent permeability of HSA647. The errors are confidence intervals from the fitting.

	P_{app} (cm/s)x10^{-6}	
Condition	w/o Inhibitor	w/ Inhibitor
Static	0.42±0.003	0.36±0.01
Pressure (0-2.5hrs)	9.01±0.258	6.86±0.51
Pressure (20-48hrs)	0.14±0.003	0.49±0.01
Flow (0-2.5hrs)	4.90±0.196	1.37±0.01
Flow (20-48hrs)	0.16±0.01	0.98±0.01

Table S4- fluorescence Endpoints after 48 hours of experiment

	AVG R123 conc. (uM) In well
static	1.1
Static inhibited	0.22
pressure	0.68
pressure Inhibited	0.24
flow	0.92
flow inhibited	0.25
